# Mono(2-ethylhexyl) phthalate induces transcriptomic changes in placental cells based on concentration, fetal sex, and trophoblast cell type

**DOI:** 10.1007/s00204-023-03444-0

**Published:** 2023-01-25

**Authors:** Samantha Lapehn, Scott Houghtaling, Kylia Ahuna, Leena Kadam, James W. MacDonald, Theo K. Bammler, Kaja Z. LeWinn, Leslie Myatt, Sheela Sathyanarayana, Alison G. Paquette

**Affiliations:** 1 Center for Developmental Biology and Regenerative Medicine, Seattle Children’s Research Institute, 1900 9th Ave, Jack R. MacDonald Building, Seattle, WA 98101 USA; 2 Department of Obstetrics and Gynecology, Oregon Health and Science University, Portland, OR 97239 USA; 3 Department of Environmental and Occupational Health Sciences, University of Washington, Seattle, WA 98195 USA; 4 Department of Pediatrics, University of Washington, Seattle, WA 98195 USA; 5 Department of Psychiatry, University of California-San Francisco, San Francisco, CA 94143 USA; 6 Center for Child Health, Behavior and Development, Seattle Children’s Research Institute, Seattle, WA 98101 USA

**Keywords:** Phthalates, Placenta, Transcriptome, Transcription factor, MEHP

## Abstract

Phthalates are ubiquitous plasticizer chemicals found in consumer products. Exposure to phthalates during pregnancy has been associated with adverse pregnancy and birth outcomes and differences in placental gene expression in human studies. The objective of this research was to evaluate global changes in placental gene expression via RNA sequencing in two placental cell models following exposure to the phthalate metabolite mono(2-ethylhexyl) phthalate (MEHP). HTR-8/SVneo and primary syncytiotrophoblast cells were exposed to three concentrations (1, 90, 180 µM) of MEHP for 24 h with DMSO (0.1%) as a vehicle control. mRNA and lncRNAs were quantified using paired-end RNA sequencing, followed by identification of differentially expressed genes (DEGs), significant KEGG pathways, and enriched transcription factors (TFs). MEHP caused gene expression changes across all concentrations for HTR-8/SVneo and primary syncytiotrophoblast cells. Sex-stratified analysis of primary cells identified different patterns of sensitivity in response to MEHP dose by sex, with male placentas being more responsive to MEHP exposure. Pathway analysis identified 11 KEGG pathways significantly associated with at least one concentration in both cell types. Four ligand-inducible nuclear hormone TFs (*PPARG*, *PPARD*, *ESR1*, *AR*) were enriched in at least three treatment groups. Overall, we demonstrated that MEHP differentially affects placental gene expression based on concentration, fetal sex, and trophoblast cell type. This study confirms prior studies, as enrichment of nuclear hormone receptor TFs were concordant with previously published mechanisms of phthalate disruption, and generates new hypotheses, as we identified many pathways and genes not previously linked to phthalate exposure.

**Supplementary Information:**

The online version contains supplementary material available at 10.1007/s00204-023-03444-0.

## Introduction

Phthalate plasticizers are ubiquitous chemicals in the man-made environment that have been linked with a host of adverse pregnancy outcomes (Lucaccioni et al. 2021). Exposure to phthalates occurs through a variety of sources including personal care products, food packaging, toys, pharmaceuticals, and medical equipment (Tuan Tran et al. 2022). Phthalates have been classified as endocrine-disrupting chemicals due to their ability to interact with hormone receptors and cause changes to hormone concentrations and activity (Sathyanarayana et al. 2014; Engel et al. 2017; Beg and Sheikh 2020). Many studies have explored the association between gestational phthalate exposure and adverse pregnancy and birth outcomes as previously reviewed (Lucaccioni et al. 2021). Specifically, prenatal phthalate exposure has been associated with perinatal health outcomes including decreased anogenital distance in male infants (Swan et al. 2005) and increased odds of preterm birth (Ferguson et al. 2014, 2019). It has also been associated with disruptions to key hormones involved in fetal reproductive development as well as the regulation of parturition, including decreased maternal serum testosterone concentration (Sathyanarayana et al. 2014), increased maternal serum estrone and estradiol concentrations (Sathyanarayana et al. 2017), decreased second trimester corticotropin-releasing hormone (Cathey et al. 2019), and altered human chorionic gonadotropin expression (Adibi et al. 2017).The placenta has been investigated as a regulator of these adverse outcomes in a number of studies, highlighting the need for a better understanding of how phthalates affect placental physiology (Warner et al. 2021).

As a fetal organ that is unique to the gestational period, the placenta plays a role in mediating outcomes of pregnancy and fetal development through nutrient and oxygen exchange as well as hormone production and signaling (Burton and Fowden 2015). The placenta is the primary barrier between mother and fetus, and protects the fetus from environmental exposures, such as smoking, air pollution, and chemicals, including endocrine disruptors like phthalates (Vrooman et al. 2016; Everson and Marsit 2018). The effects of maternal phthalate exposure on the placenta have been extensively studied in humans, animals, and cells as reviewed by Warner et al. and Strakovsky and Schantz (Strakovsky and Schantz 2018; Warner et al. 2021). Most of these studies evaluate phthalates by studying their metabolites, as phthalate parent compounds are quickly degraded through a two-step metabolism involving hydrolysis and conjugation followed by excretion in urine (Frederiksen et al. 2007). Due to the rapid metabolic transformation of phthalates, the monoester and subsequent metabolites are the primary species that the fetus is exposed to and believed to cause adverse effects in humans (Zhang et al. 2021). The initial hydrolysis steps of phthalate metabolism are carried out by lipase and esterase enzymes in the intestine and parenchyma, so most in vitro studies of placental exposure to phthalates utilize the metabolites rather than the parent compound (Frederiksen et al. 2007; Strakovsky and Schantz 2018). In addition to being measurable in human urine, phthalates have also been recorded in maternal and cord blood indicating that phthalates are able to cross the placenta and enter fetal circulation (Latini et al. 2003; Li et al. 2013; Maekawa et al. 2017; Caserta et al. 2018). Studies that have directly measured phthalates in maternal and fetal placental perfusate (Mose et al. 2007) or placental tissue (Poole and Wibberley 1977) have also concluded that phthalates can cross the placenta.

RNA sequencing is a discovery-based methodology that can reveal genes and pathways perturbed by environmental exposures and be used to study relationships between prenatal exposures and birth or later life health outcomes (Lapehn and Paquette 2022). Recently, we evaluated the maternal urinary concentrations of 16 phthalate metabolites in the second and third trimester of pregnancy with the placental transcriptome at birth in the CANDLE study, a cohort based in Memphis, TN (*N* = 760). This study identified 38 differentially expressed genes (DEGs) associated with four phthalate metabolites across the second and third trimester, as well as several fetal sex-specific gene and phthalate associations (Paquette et al. 2021). To date, this has been one of only two studies evaluating the association between urinary phthalate concentrations during pregnancy and placental mRNA or long non-coding RNA (lncRNA) in placentas at birth in humans (Machtinger et al. 2018; Paquette et al. 2021). Mono(2-ethylhexyl) phthalate (MEHP), the most commonly studied metabolite of di(2-ethylhexyl)phthalate (DEHP), showed the highest number of associations with gene expression including ten lncRNAs in Machtinger et al. (Machtinger et al. 2018). We also identified several sex-specific associations in gene expression in relation to maternal urinary concentrations of MEHP in our prior analysis of phthalate metabolites and the placental transcriptome in the CANDLE study (Paquette et al. 2021). Though these two studies provide strong evidence for phthalate-induced gene expression changes in the placenta, the observational nature of the studies does not allow for causal conclusions. While there have been several in vitro studies on the effects of phthalates on the placenta during pregnancy, most of these studies have been limited in deriving mechanisms of toxicity due to evaluating expression of only a small subset of candidate genes (Tetz et al. 2013, p. 201; Wang et al. 2016; Meruvu et al. 2016b; Adibi et al. 2017; Strakovsky and Schantz 2018; Zhang et al. 2020a; Warner et al. 2021). To date, there has only been a single in vitro study utilizing RNA sequencing technology to evaluate the effect of phthalates in placental cells which used trophoblast stem cells from a rhesus monkey, limiting translation of the results to human pregnancies (Midic et al. 2018).

Because epidemiological assessment of the placenta is most commonly performed within bulk placental tissue, in vitro assessment of the placenta presents a unique opportunity to assess the cell type-specific responses to environmental exposures, since the placenta is a heterogenous tissue comprising multiple trophoblast cell types with distinct functions in placental physiology. Cytotrophoblasts (CTBs) and syncytiotrophoblasts (STBs) are both villous trophoblast cells with CTBs serving as precursors that develop into multi-nucleated STBs, which reside as the outer layer of the placental villi where they act as the primary exchange surface of the placenta (Farah et al. 2020). Extravillous trophoblasts (EVTs) are an invasive trophoblast cell type that embed the placenta in the decidual wall and assist in spiral artery remodeling (Farah et al. 2020). This research aims to expand upon the knowledge of phthalate metabolite-induced transcriptome changes in the placenta from human studies by evaluating the causality between phthalate exposure and gene expression changes using in vitro methodology. This study performed RNA sequencing following exposure to phthalate metabolite MEHP, in both immortalized (HTR-8/SVneo) and primary placental cells that are representative of two different placental cell types and trimesters of origin (HTR-8/SVneo: 1st trimester EVTs and primary cells: term syncytiotrophoblasts). We also compare the findings of our differential gene expression analysis and pathway analysis to the results of the CANDLE study to identify similarities in phthalate-induced differences between bulk placental tissue and in vitro models of the placenta. Results of this work will advance knowledge of phthalate-induced changes to the placental transcriptome, while providing additional evidence for cell type-specific responses that cannot be easily assessed in human studies of bulk placental tissue.

## Methods

### Cell culture

The first trimester EVT cell line, HTR-8/SVneo, was obtained from ATCC (#CRL-3271, batch: 70016636, obtained: January 2021). HTR-8/SVneo cells were cultured at 37 °C with 5% CO_2_ and ambient O_2_ in six-well tissue culture dishes between passages 4–7 using RPMI-1640 with L-glutamate supplemented with 10% fetal bovine serum (FBS), 1% penicillin–streptomycin (P–S), 1 mM sodium pyruvate, and 10 mM HEPES. The HTR-8/SVneo cells were grown to at least 60% confluence prior to treating with MEHP.

Placental villous tissue samples were derived from non-pathological term pregnancies (> 37 weeks gestation) from women who delivered via elective cesarean section in the absence of labor from the Labor and Delivery Unit at Oregon Health & Sciences University (OHSU). Exclusion criteria included maternal BMI > 25, maternal age < 18 or > 40 years, any current pregnancy complications (preeclampsia, gestational diabetes, or chorioamnionitis) and current smokers. Placentas were collected and weighed immediately following cesarean section. Five random samples of tissue (~ 80 g) were collected from each placenta and stored in PBS. The chorionic plate and decidua were removed from each placental sample, leaving only villous tissue, which was thoroughly rinsed in PBS to remove excess blood. Primary cytotrophoblasts were isolated from villous tissue using a protocol adapted from Eis et al*.* using trypsin/DNAse digestion, followed by density gradient purification (Eis et al. 1995). Isolated cytotrophoblast cells (5–10 × 10^6^ cells /ml) were then frozen in freezing media (10% DMSO in FBS) and stored in liquid nitrogen. Placentas were collected into a tissue repository under a protocol approved by the OHSU Institutional Review Board with informed consent from the patients. All tissues and clinical data were de-identified before being made available to the investigative team.

Primary cytotrophoblast cells were cultured in IMDM with 10% FBS and 1% penicillin/streptomycin. Cells were plated at ~ 1.5 × 10^6^ cells per well in 12-well plates and given 24 h to adhere prior to changing the media to remove non-adherent cells. Syncytialization occurs spontaneously and was confirmed under a light microscope at 48 h (Online Resource 1). Primary syncytiotrophoblast cells were incubated at 37 °C with 5% CO_2_ and ambient O_2_ in 12-well plates.

### Phthalate metabolite treatment

MEHP (Sigma Aldrich; St. Louis, MO) was prepared in 100% DMSO as a 180 mM stock solution that was stored at  – 20 °C prior to treatment. HTR-8/SVneo and primary trophoblast cells were each treated with DMSO (0.1%) or one of three final concentrations (1 µM, 90 µM, and 180 µM) of MEHP with three replicates per concentration. For the primary syncytiotrophoblast cells, cells from three male and three female placentas were used per treatment group with each replicate representing the placenta of a unique individual. There were no statistical differences in maternal BMI, maternal age, or gestational length between male and female samples (Table 1). Cells were incubated with dosed media for 24 h at 37 °C. MEHP was added to cultures at the 48 h time point and incubation continued for an additional 24 h at 37 °C before removal of media and isolation of RNA from cells.

**Table 1:** Maternal BMI, maternal age, and gestational length for primary syncytiotrophoblast samples

	Male	Female
Maternal BMI (kg/m^2^)	20.7, 22.3, 24.2	22.6, 23.3, 24.0
Maternal age (years)	32.0, 35.0, 39.0	32.0, 35.0, 35.0
Gestational length (weeks)	37.9, 38.1, 39.1	39.0, 39.0, 39.0

### RNA isolation, library preparation, and sequencing

RNA from HTR-8/SVneo cells was isolated by a phenol:chloroform extraction using TRIzol. Primary trophoblast RNA isolation was performed with the Zymo Direct-zol RNA Miniprep kit (Zymo Research, Irvine, CA), following the manufacturer’s instructions after lysing cells in 350µL TRIzol. Library preparation and paired-end RNA sequencing were performed by *Novogene, Inc.* (Beijing, China). Novogene prepared libraries with high-quality RNA after measuring the RNA integrity number (RIN) > 7 with the Agilent High-Sensitivity RNA Screentape Assay (Agilent Technologies; Santa Clara, CA). Sample library preparation was performed using the SMARTer Stranded Total RNA-Seq Kit v2 (Takara Bio Inc.; Kusatsu, Japan) for primary syncytiotrophoblast cells, followed by paired-end sequencing (150BP) using an Illumina HiSeq with a read depth of 30 million read pairs/sample. Library preparation for HTR-8/SVneo cells was performed using an optimized NEBNext Ultra II kit, followed by paired-end sequencing (150BP) with a NovaSeq 6000 and a read depth of 30 million read pairs/sample.

### RNA sequencing analysis

Transcript abundances were estimated using the pseudo-alignment program kallisto with bias correction (Bray et al. 2016) and condensed to Ensembl Gene IDs using tximport (Soneson et al. 2015). Filtering was performed to remove genes with a mean log CPM < 0, resulting in a final dataset which included 13,379 protein coding and lncRNA transcripts for HTR-8/SVneo, 15,784 transcripts for the combined primary syncytiotrophoblasts, 15,182 transcripts for male primary syncytiotrophoblasts, and 16,358 transcripts for female primary syncytiotrophoblasts. Normalization was performed using the trimmed mean of M-values (Robinson and Oshlack 2010). Differentially expressed genes between treatment groups compared to DMSO controls were identified using generalized linear models implemented within edgeR (Chen et al. 2016). Dispersion parameters were estimated using the Cox–Reid method, and then differentially expressed genes were identified using the quasi-likelihood F-tests, which is more robust and produces more reliable error rates when the number of replicates is small (Lun et al. 2016). Regression models for primary cells included precision variables such as RNA integrity number (RIN) and Sample ID, which minimized interindividual variation and sex differences in our full model (not stratified for sex). Genes were considered differentially expressed at a Benjamini–Hochberg false discovery rate (FDR) < 0.05. We also compared the genes that were differentially expressed in our cell models to lists of genes that are over-expressed in the placenta or specific to the placenta via the Human Protein Atlas which categorizes genes based on their degree of tissue specificity or over-expression (Uhlén et al. 2015).

### Pathway analysis

Pathway analysis was performed using the self-contained gene set testing method Fry to identify non-disease-associated pathways from the Kyoto Encyclopedia of Genes and Genomes (KEGG) database (Kanehisa and Goto 2000; Wu et al. 2010). Pathways with FDR < 0.05 were considered significant.

### Transcription factor enrichment analysis

Transcription factor (TF) enrichment analysis was performed using Enrichr with the ENCODE/ChEA consensus TFs from ChIP-X (Chen et al. 2013; Kuleshov et al. 2016; Xie et al. 2021). TFs with an FDR < 0.05 were considered significant.

## Results

We treated two placental cell lines (Primary and HTR-8/SVneo cells) with three different concentrations of MEHP based on a comprehensive review of existing literature surrounding phthalates in the placenta. HTR-8/SVneo was selected for use in this study because it is not derived from a choriocarcinoma, and it represents an extravillous trophoblast phenotype that differs from the other primary syncytiotrophoblast model utilized in this study. A literature review of phthalate exposure in placental cell lines revealed that HTR-8/SVneo is one of the most commonly used cell lines for this work, thus increasing cross-study comparability (Online Resource 2) (Xu 2005; Xu et al. 2006, 2016; Tetz et al. 2013, 2015; Wang et al. 2016; Meruvu et al. 2016a, b; Pérez-Albaladejo et al. 2017; Gao et al. 2017; Petit et al. 2018; Shoaito et al. 2019; Zhang et al. 2020b; Du et al. 2020)*.* To reduce the influence of gestational age and labor status on gene expression in the primary placental cells, we only used cells derived from full-term (> 37 weeks) deliveries by cesarean section. Our sex-stratified analysis was roughly matched on maternal age and BMI showing no significant differences across groups. 90 µM and 180 µM MEHP concentrations were selected to be comparable to previous studies of MEHP exposure in placental cells (Xu 2005; Tetz et al. 2013; Wang et al. 2016; Meruvu et al. 2016a, b; Gao et al. 2017), while the lower MEHP concentration (1 µM) was within the range of MEHP measured in maternal urine in the CANDLE study (1.1 × 10^–4^ µM to 2.2 µM) (Paquette et al. 2021). The selected concentrations are also in alignment with a recent assessment of placental phthalate concentrations in the CANDLE study, which reported mean placental MEHP concentrations of 20.4 µM in a subset of CANDLE participants (N = 50) (Liang et al. 2022).

### Differentially expressed genes

**Fig. 1:**
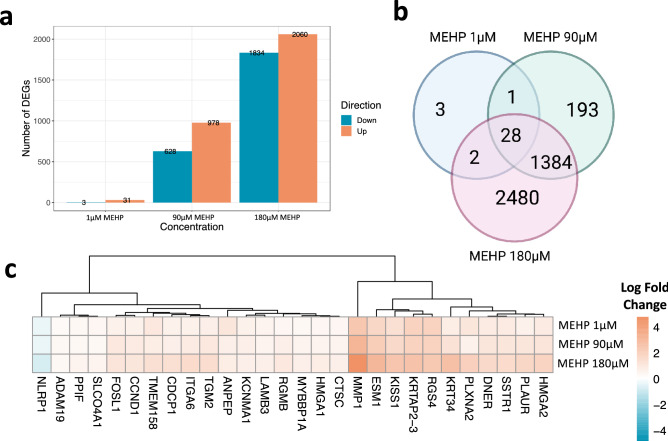
HTR-8/SVneo differentially expressed genes (DEGs). **a** Number and directionality of DEGs (FDR < 0.05) for MEHP 1 µM, 90 µM, and 180 µM in HTR-8/SVneo cells (N = 3/concentration). **b** Venn diagram showing DEG overlap across MEHP concentration groups (FDR < 0.05) in HTR-8/SVneo cells. Created with Biorender.com. **c** Heatmap of log-fold change (LogFC) for 28 shared DEGs across MEHP 1 µM, 90 µM, and 180 µM concentrations

MEHP treatment in HTR-8/SVneo EVT cells induced a higher number of differentially expressed genes (DEGs, FDR < 0.05) with increasing MEHP concentrations (Fig. 1a). In total, there were 34 DEGs for 1 µM MEHP, 1606 DEGs for 90 µM MEHP, and 3894 DEGs for 180 µM. 63 of the 4091 HTR-8/SVneo DEGs were considered placenta specific, enhanced or enriched, based on the human protein atlas (Online Resource 3). One placenta-enhanced gene (*KISS1*) was significantly increased across all three MEHP concentrations in the HTR-8/SVneo cell line and was also significant, but with decreased expression at the 180 µM MEHP concentration in the primary cells. Across all three concentrations of MEHP treatment, there were more genes with increased expression than decreased expression compared to DMSO controls. 28 genes were significantly affected by all MEHP concentrations (Fig. 1b). Of these genes, 27 were upregulated, with 18 genes exhibiting a positive dose response (Fig. 1c). Across the DEGs identified at each concentration, 19 lncRNAs were altered with 90 µM treatment, and 77 lncRNAs were altered with 180 µM MEHP treatment.

**Fig. 2:**
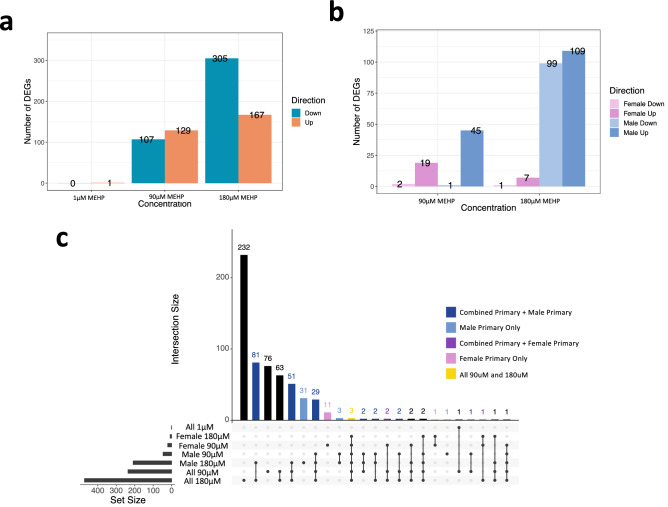
Primary syncytiotrophoblast differentially expressed genes (DEGs). **a** Number and directionality of DEGs (FDR < 0.05) for MEHP 1 µM, 90 µM, and 180 µM in primary syncytiotrophoblast cells (N = 6/concentration). **b** Number and directionality of DEGs (FDR < 0.05) for MEHP 1 µM, 90 µM, and 180 µM in primary syncytiotrophoblast cells stratified by sex (female *N* = 3/concentration, male *N* = 3/concentration). **c** UpSet plot showing the overlap of DEG groups across full primary syncytiotrophoblast data (*N* = 6 90 µM and 180 µM, *N* = 5 1 µM) and sex-stratified primary syncytiotrophoblast data (female *N* = 3, male *N* = 3)

Primary syncytiotrophoblasts were assessed for DEGs in a combined (*N* = 6) and sex-stratified analysis (*N* = 3 Male, 3 Female). Overall, 552 unique DEGs were associated with at least one of three MEHP concentrations, and 55 of these DEGs were considered enriched, enhanced, or specific to the placenta based on the human protein atlas. Of particular note, there were four genes expressed only in the placenta (based on the HPA) that were significantly decreased by at least one concentration of MEHP in the primary cells including *PSG3*, *PSG9*, *LGALS13*, and *PSG8* (Online Resource 3). In the combined analysis, there was also a higher number of DEGs with increasing MEHP concentration (Fig. 2a). The sex-stratified analysis revealed dimorphisms in transcriptional response to MEHP, with male samples having a higher number of DEGs with increased MEHP concentration, while female samples showed a non-monotonic dose response with the highest number of DEGs in the 90 µM group (Fig. 2b). Comparison of DEGs across the combined and sex-stratified analysis revealed 35 DEGs significant only within the male-stratified analysis and 12 DEGs significant only within the female-stratified analysis (Fig. 2c). Three genes (*FABP4*, *STRIP2*, *HMGCS2*) were significantly altered by 90 µM and 180 µM concentrations in the combined and both sex-stratified analyses (Fig. 2c). After treatment with 90 µM MEHP, 11 lncRNAs were statistically significant in the combined analysis and 2 in the male-specific analysis, while after treatment with 180 µM MEHP 7 lncRNAs were statistically significant in the combined and 1 lncRNA in the male-specific analysis. A list of DEGs from each cell type are available in Online Resource 3.

### Pathway analysis

**Fig. 3:**
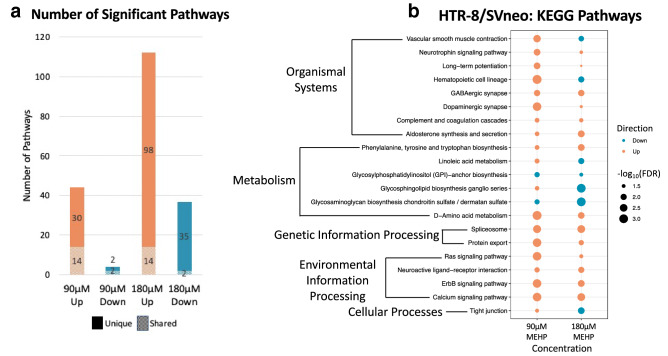
HTR-8/SVneo pathway analysis performed by self-contained gene set testing with Fry using KEGG pathways. **a** Number and directional concordance of significant (FDR < 0.05) KEGG pathways. Pathways shared across multiple concentrations are indicated by striped shading and pathways unique to a single concentration are indicated by solid shading. Orange bars represent increased pathways and teal bars represent decreased pathways. **b** Shared KEGG pathways between MEHP 90 µM and MEHP 180 µM annotated by KEGG categories

**Fig. 4:**
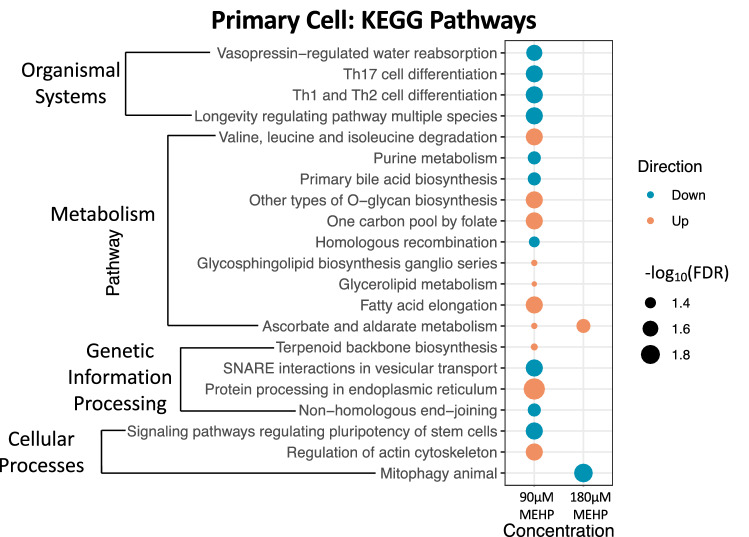
Significant (FDR < 0.05) KEGG pathways for MEHP 90 µM and 180 µM identified by self-contained gene set testing with Fry for all primary syncytiotrophoblast cells

**Fig. 5:**
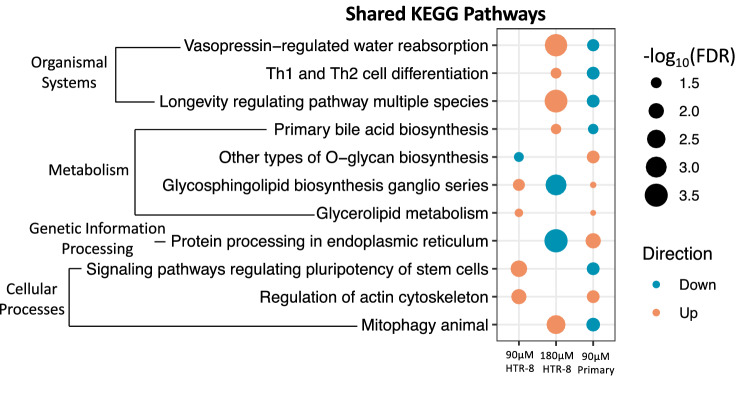
Shared KEGG pathways in HTR-8/SVneo and primary syncytiotrophoblast cells identified through self-contained gene set testing with Fry that were significant (FDR < 0.05) for at least one MEHP concentration in each cell type

Pathway analysis using rotational gene set testing identified 174 significant (FDR < 0.05) KEGG pathways for 90 µM and 180 µM MEHP across HTR-8/SVneo and primary syncytiotrophoblast cells. Most pathways were significant only within a single dose group (Fig. 3a). In HTR-8/SVneo cells, there were 21 pathways that were significant following both 90 µM and 180 µM MEHP treatment (Online Resource 4), with 16 pathways showing directional concordance (Fig. 3b). The calcium signaling pathway was increased for both concentrations and had the lowest average FDR value. Pathway analysis of primary syncytiotrophoblast cells identified 20 significant pathways for 90 µM MEHP and 2 significant pathways for 180 µM MEHP with a single pathway—ascorbate and aldarate metabolism, significant across the two MEHP concentrations (Fig. 4). A single pathway—terpenoid backbone synthesis—was significantly increased following 180 µM MEHP treatment in the male samples only. Comparing the significant KEGG pathways between HTR-8/SVneo and primary syncytiotrophoblast cells, there were 11 pathways that were significantly altered in relation to at least one MEHP concentration in each cell type (Fig. 5). Of these shared pathways, two (glycerolipid metabolism and regulation of actin cytoskeleton) showed directional concordance across cell types and concentrations.

### Transcription factor analysis

**Fig. 6:**
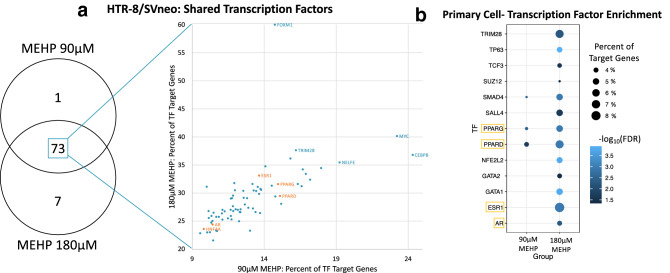
Transcription factor enrichment analysis by Enrichr using the ENCODE/ChEA consensus TF library for HTR-8/SVneo (**a**) and primary syncytiotrophoblast cells (**b**). **a** Scatterplot of shared TFs for MEHP 90 µM and 180 µM in HTR-8/SVneo cells plotted by percent of downstream gene targets that were identified as significant for each corresponding TF. Orange labeled TFs are nuclear hormone receptors. **b** Bubble plot of all significant (FDR < 0.05) for primary syncytiotrophoblast TFs

Transcription factor (TF) enrichment analysis was performed using Enrichr, with TFs characterized from the ENCODE/ChEA consensus TF library. Enrichr identified potential TF regulators of our DEG lists by performing an overrepresentation test based on TF binding sites within the target gene list. In HTR-8/SVneo cells, 74 TFs were enriched for DEGs associated with 90 µM MEHP treatment and 80 TFs were significantly enriched for DEGs associated with 180 µM MEHP treatment (Fig. 6a). There were 73 TFs that were significantly enriched from both concentrations, with 5 TFs (*HNF4A*, *AR*, *ESR1*, *PPARG*, *PPARD*) defined as ligand-inducible nuclear hormone receptors, based on the IUPHAR/BPS Guide to Pharmacology (Alexander et al. 2021). The top three significant TFs were *MAX*, *MYC*, and *SIN3A* for 90 µM MEHP and *MAX*, *MYC*, and *NFYB* for 180 µM MEHP. A full list of TFs significantly enriched within the HTR-8/SVneo cell analysis can be found in Online Resource 5. In primary syncytiotrophoblasts, 3 TFs were significantly enriched for 90 µM MEHP and 13 were significantly enriched for 180 µM MEHP (Fig. 6b). In the sex-stratified analysis, no TFs were significantly enriched following MEHP treatment in females. The male-stratified analysis, however, identified two significant TFs (*PPARG*, *PPARD*) enriched for 90 µM MEHP and six TFs (*NFE2L2*, *PPARG*, *TCF3*, *SALL4*, *GATA1*, *SMAD4*) enriched for genes treated with 180 µM MEHP. There were no TFs enriched after treatment with 1 µM MEHP in either cell type.

**Fig. 7:**
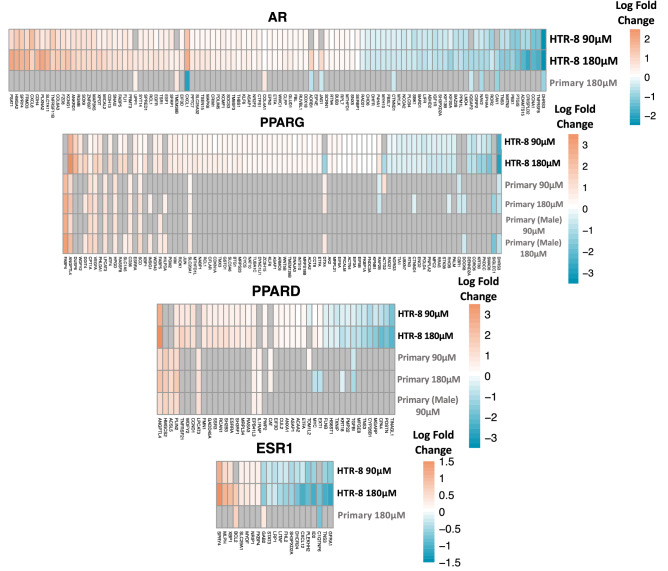
Heatmap of the LogFC for significant (FDR < 0.05) downstream genes of enriched nuclear hormone receptor TFs (ESR1, PPARG, PPARD, AR) that were in at least two cell/concentration groups. Gray-shaded cells are genes that were not significant for that treatment group and cell line. The full list of genes is presented in Online Resource 3

Four ligand-inducible nuclear hormone receptor TFs (*PPARD*, *PPARG*, *ESR1*, *AR*) were significantly enriched following MEHP treatment for at least one MEHP concentration in both cell types. *PPARG* and *PPARD* were also enriched for at least one MEHP concentration in the male-stratified analysis. Changes in gene expression associated with DEGs downstream of these TFs in at least two cell type or concentration groups are shown in Fig. 7. *PPARG*, *PPARD*, and *AR* primarily regulated genes which had increased expression after treatment with MEHP, while *ESR1* regulated genes which had decreased expression after treatment with MEHP.

### Comparison to the CANDLE cohort

In our previous evaluation of associations between the placental transcriptome and phthalate metabolites in the CANDLE cohort (*N* = 760), 12 genes were significantly associated (FDR < 0.05) with the second trimester urinary MEHP concentrations in males alone (Paquette et al. 2021). Three of these 12 genes (*NEAT1*, *ANKRD10*, *PHLDB2*) were also significantly associated with MEHP treatment in HTR-8/SVneo and/or primary syncytiotrophoblast cells in this in vitro study of MEHP. There were no KEGG pathways that were associated with MEHP after adjustment for multiple comparisons, but there were 20 pathways associated with MEHP that were marginally significant with an unadjusted *p* value less than 0.05 (Online Resource 6). Comparing the pathway analysis results of our two cell lines to those from CANDLE, we found that of the 20 pathways (*p* < 0.05) for the CANDLE MEHP data, 17 were fully significant (FDR < 0.05) in our analysis in relation to at least one MEHP concentration in HTR-8/SVneo and/or primary syncytiotrophoblast cells. Two of these pathways (Vasopressin-regulated water reabsorption and mitophagy) were found in at least one CANDLE time point and one MEHP concentration for each cell line. For both pathways, there was decreased directional concordance for the CANDLE samples and primary syncytiotrophoblasts, while HTR-8/SVneo cells showed increased expression for these pathways, highlighting potential differences across placental trophoblast subtypes.

## Discussion

Phthalate metabolite MEHP’s effects on the placenta have been extensively studied through epidemiological work and candidate gene expression studies; however, only recently have MEHP’s global effects across the placental transcriptome been evaluated through RNA sequencing. To date, there has only been one study that assessed the complete mRNA and lncRNA placental transcriptome in a large human population (Paquette et al. 2021). To our knowledge, the current in vitro study represents the first transcriptome-wide study of the placental response to MEHP using commercial and primary placental cell lines. Through this study, we identified DEGs following exposure to three concentrations of MEHP in two cell types, evaluated the effect of fetal sex on gene expression in primary syncytiotrophoblast cells, contextualized the biological context of DEGs through pathway analysis, and investigated upstream causes of gene expression changes through transcription factor enrichment analysis. Our results highlight that MEHP exposure causes gene expression changes that are unique to MEHP concentration, fetal sex, and placental cell type.

MEHP exposure caused substantial changes in gene expression in both HTR-8/SVneo and primary syncytiotrophoblasts with 4,091 and 552 total genes that were affected in each respective cell type. In HTR-8/SVneo cells, there were 28 genes that were significantly affected by MEHP treatment at all three concentrations with all but one (*NLRP1*) exhibiting increased expression. *NLRP1* has not previously been associated with MEHP exposure, but the *NLRP1* inflammasome has been implicated as part of a combined inflammation/autophagy response to oxidative stress in HTR-8/SVneo cells (Li et al. 2021). Placental exposure to MEHP or its parent compound DEHP have both been linked with a variety of oxidative stress end points indicating a potential link between MEHP and *NLRP1* expression (Martínez-Razo et al. 2021). The three genes with the highest average log fold change after MEHP exposure were *MMP1*, *ESM1*, and *KRTAP2-3*. These three genes, which were not significant in the primary syncytiotrophoblast cells, may be related to extravillous trophoblast cell function captured within the HTR-8/SVneo cells. Two important functions of EVT cells during the first trimester of pregnancy include invasion of the maternal decidua and spiral artery remodeling (Burton and Jauniaux 2015). *MMP1* is a matrix metalloproteinase that is involved in the breakdown of extracellular matrices, which may help EVTs to move and embed themselves within the decidual wall (Chahar et al. 2021). *KRTAP2-3* is a keratin-associated protein. Keratins are intermediate filament components of the cytoskeleton that help EVT cells invade the decidua and the spiral arteries (Gauster et al. 2013). *ESM1* is an endothelial cell-specific molecule, frequently termed endocan that is involved in the process of angiogenesis. Although a specific angiogenic role for *ESM1* in the placenta is not known, it could be involved in spiral artery remodeling and its expression levels have been associated with numerous adverse pregnancy outcomes including preeclampsia and gestational diabetes (Chang et al. 2015; Hentschke et al. 2015; Murthi et al. 2016; Cross et al. 2022). Although none of these three genes have previously been associated with MEHP exposure, expression of *MMP-9,* another member of the matrix metalloproteinase family, was decreased following MEHP exposure in HTR-8/SVneo cells (Gao et al. 2017).

In both cell types, the 1 µM MEHP concentration caused the smallest number of gene expression changes; however, not all the genes that were significantly changed at 1 µM were significantly affected in the higher dose groups. In HTR-8/SVneo, there were two genes (*DHRS2* and *TASOR2*) that were only significant after 1 µM and 180 µM MEHP exposure, while one gene (*IGFBP5*) was only significant in the two lower concentrations (1 µM and 90 µM). *IGFBP5* encodes a binding protein of insulin-like growth factors which have been previously demonstrated to promote trophoblast proliferation and invasion of the maternal decidua by EVTs (Crosley et al. 2014). Though there are no studies of MEHP’s effect on *IGFBP5* expression, a study of chronic exposure to MEHP’s parent compound, DEHP, performed in *Rhesus macaque* embryonic stem cells found that DEHP exposure increased the expression of *IGFBP5* which matches the directionality of the response seen in the HTR-8/SVneo cells in our study (Midic et al. 2018). Interestingly, all the genes discussed here were directionally concordant for the concentrations where they showed significance and were only significant in one of the two cell types analyzed.

We compared the results of the in vitro evaluation of MEHP on the placenta to the prior epidemiological analysis of phthalates on the placenta completed in the CANDLE study (Paquette et al. 2021). The lncRNA *NEAT1* was significantly decreased after 90 µM and 180 µM MEHP treatment in HTR-8/SVneo cells as well as increased in the male-specific analysis of the primary syncytiotrophoblasts at 90 µM MEHP. *NEAT1* expression in the placenta has previously been associated with other phthalate metabolites, including related DEHP metabolites mono(2-ethyl-5-oxohexyl) phthalate (MEOHP) and mono-(2-ethyl-5-carboxypentyl) phthalate (MECCP) as well as mono(carboxyisooctyl) phthalate (MCIOP) and monomethyl phthalate (MMP), suggesting a potential role for this lncRNA in phthalate-induced gene expression disruption (Machtinger et al. 2018; Paquette et al. 2021). Two additional genes, *ANKRD10* and *PHLDB2*, were significantly decreased following 180 µM MEHP treatment in HTR-8/SVneo cells. For both of these genes. the CANDLE study was their first known association with MEHP exposure (Paquette et al. 2021). None of the other genes significantly associated with MEHP from the CANDLE study were altered after MEHP treatment in the combined or female-specific primary syncytiotrophoblasts, suggesting that the effects we observe may be fetal sex and placental cell type specific.

Results of our analysis were also compared to other in vitro studies of MEHP in HTR-8/SVneo or primary syncytiotrophoblast cells. In HTR-8/SVneo cells exposed to 1–200 µM MEHP for 24 h by Gao et al., the activity of *MMP9* was decreased (100 and 200 µM) and the protein expression of *TIMP-1* was increased (10, 100, 200 µM); however, the mRNA expression levels of both *MMP9* and *TIMP-1* were not changed (Gao et al. 2017). In our HTR-8/SVneo cells, we noted an increase in *TIMP-1* expression (90 and 180 µM) and a decrease in *MMP9* expression (180 µM) mirroring the directionality of the activity and protein expression changes from Gao et al. Neither of these genes were affected by MEHP exposure in the primary syncytiotrophoblast cells. Another study of MEHP exposure in HTR-8/SVneo cell line found increased expression of *PTGS2*; however, these results were not replicated in our study (Tetz et al. 2013). A study of MEHP exposure in primary syncytiotrophoblast cells identified increased expression of *CRH* and *COX-2*, but this finding was also not replicated in either of our cell lines (Wang et al. 2016).

Fetal sex is an important biological variable in placental omics analyses, and it has been shown to affect gene expression following phthalate exposure (Paquette et al. 2021). In the CANDLE phthalate study, 14 genes with differential expression in females were associated with five phthalate metabolites and 25 genes in males were associated with five phthalate metabolites and DEHP, with MEHP having the highest number of sex-specific findings with 12 associations identified in males (Paquette et al. 2021). The identification of sex-specific differences underscores the importance of considering fetal sex as a contributing variable in analyses of environmental exposures. Using primary syncytiotrophoblast cells presented in this study provides a unique opportunity to assess fetal sex in an in vitro cell model of the placenta and phthalate exposure.

Our study identified 35 genes in male placentas and 12 genes in female placentas that were exclusive to the sex-stratified analysis and not present in the full model for primary syncytiotrophoblasts. Of these unique genes in males, there were three (*FABP5*, *MGAT3*, *NHS*) that were significant for both 90 µM and 180 µM concentrations, while female placentas had a single gene (*LRP1B*) that was significant for both concentrations. While none of these genes have previous associations with MEHP, *FABP5* has been associated with DEHP exposure in mice and HepG2 cells, as well as a DEHP-containing phthalate mixture in rats (Stenz et al. 2017; Wei et al. 2017; Scarano et al. 2019). Placental DNA methylation levels of *LRP1B*, which was significant only in female primary cells, and codes for a low-density lipoprotein receptor, had previously been linked with gestational diabetes mellitus and maternal glucose levels (Houde et al. 2015). Three genes (*FABP4*, *STRIP2*, and *HMGCS2*) were significant at 90 µM and 180 µM MEHP in all three primary syncytiotrophoblast models (male, female, and combined). Of these three genes, two (*FABP4* and *HMGCS2*) are involved in the mechanisms of fatty acid use and the third (*HMGCS2*) in cytoskeletal organization. In mouse stromal and fat cells, expression of *FABP4* increases following MEHP exposure, which matches the directionality of gene expression change seen in the primary syncytiotrophoblast cells (Watt and Schlezinger 2015; Chiang et al. 2016). *STRIP2* and *HMGCS2* expressions have not previously been associated with MEHP exposure, but expression of genes was decreased in human embryonic stem cells following DEHP exposure, and *HMGCS2* expression increased after DEHP treatment in mouse liver tissue (Eveillard et al. 2009; Fang et al. 2019).

Identifying transcriptomic differences in the response of male and female placentas to MEHP supports previous research on sex differences in response to phthalates and sex differences in placental adaptation mechanisms. Some of the first widely noted anatomical effects of phthalates were reduced anogenital distance and incomplete testicular descent in male infants (Swan et al. 2005). Since then, additional recent sex-specific findings of phthalate exposure have included differences in body and organ weight in weanling mice exposed prenatally to phthalates (Neier et al. 2019), as well as differences in associations of adverse birth outcomes with mixtures of phthalate metabolites in the PROTECT cohort (Cathey et al. 2022). The placenta is a particularly relevant organ for identifying sex-specific differences in environmental exposures as there are already known differences in the ratio of male vs female fetuses compared to their placental weight, with male fetuses having comparatively smaller placentas with lower reserve capacity than female fetuses, which is understood to be the result of males prioritizing in utero growth more than females (Eriksson et al. 2010; Meakin et al. 2021). This discrepancy in placental reserve capacity could therefore cause some of the sex-specific effects seen in the placenta when faced with environmental exposures, such as phthalates.

In HTR-8/SVneo cells, 21 pathways were significantly enriched for genes whose placental expression was altered after both 90 µM and 180 µM MEHP. Of these shared pathways, the top three (based on average FDR) were calcium signaling pathway, spliceosome, and ErbB signaling pathway, all of which exhibited directional concordance for the two dose groups with increased pathway expression. Although there is no published research linking these pathways to MEHP exposure, calcium signaling pathway and ErbB signaling pathway were both enriched for genes reported to be associated with MEHP in the Comparative Toxicogenomics Database (CTD) (Davis et al. 2021). In primary syncytiotrophoblast cells, there was only a single pathway, ascorbate and aldarate metabolism that was identified for both 90 µM and 180 µM MEHP concentrations. This pathway was also significantly enriched for MEHP-associated genes in the CTD, but not otherwise linked to MEHP exposure in the currently available literature (Davis et al. 2021).

Across both cell types, there were 11 pathways that were significantly associated with at least one MEHP concentration. Only two of these pathways, glycerolipid metabolism and regulation of actin cytoskeleton, exhibited directional concordance with both pathways having increased expression. Neither pathway had previous experimental evidence of a connection with MEHP, but regulation of actin cytoskeleton was enriched for MEHP-associated genes in the CTD (Davis et al. 2021). The two pathways that were identified for at least one concentration in HTR-8/SVneo, primary syncytiotrophoblasts, and the CANDLE study (vasopressin-regulated water reabsorption and mitophagy) had not previously been associated with MEHP exposure prior to the CANDLE study (Paquette et al. 2021). The actin cytoskeleton is involved in the processes of invading and anchoring the placenta to the decidual wall as well as the syncytialization of villous trophoblasts all rely on cytoskeletal reorganization (Rote et al. 2010; Farah et al. 2020). Alterations in the regulation of the actin cytoskeleton could thus result in impaired placental development and function. Glycerolipid metabolism is also an essential placental process with evidence that lipid accumulation above normal physiological levels induces lipid droplet formation, increases cytokine production, and causes changes to syncytialization and hormone production in the placenta (Pathmaperuma et al. 2010). Of the pathways shared with the CANDLE study (Online Resource 6), the tight junction pathway which was significant in HTR-8/SVneo cells is of particular interest, as tight junctions are a critical component of trophoblast cell function with roles in differentiation of cytotrophoblasts to extravillous trophoblasts or syncytiotrophoblasts, as well as involvement in extravillous trophoblast invasion of the maternal decidua (Adu-Gyamfi et al. 2021). Phthalate disruption of tight junctions has been most extensively studied in testes with a focus on Sertoli cells (Zhang et al. 2008; Sobarzo et al. 2009, 2015; Hu et al. 2014; Kumar et al. 2015). Therefore, this study and the CANDLE study are two of the first to indicate a potential role for phthalate disruption of tight junctions in the placenta.

With knowledge of genes that are altered by MEHP exposure, it is next imperative to understand the upstream cause of the phthalate-induced gene expression changes. Phthalates are hypothesized to affect gene expression through direct binding to nuclear steroid hormone receptors which is attributable to the similarity of the phthalate metabolite benzene ring which mimics Ring A of steroid hormone structures (Baker 2014; Beg and Sheikh 2020). Specifically, phthalates may bind to the androgen receptor (AR), estrogen receptors (ERs) and peroxisome proliferator-activated receptors (PPARs), which in addition to being nuclear hormone receptors are also ligand-inducible transcription factors (Engel et al. 2017; Beg and Sheikh 2020). Metabolites of DEHP (including MEHP) have been shown to activate *PPARA* and *PPARG*, but do not affect the activity of *AR* or *ERα* or *ERß* (Engel et al. 2017). In this study, MEHP did not alter the activity of *AR* or *ER*, but DEHP exposure did inhibit the activity of all three of these receptors (Engel et al. 2017). In the current study, we identified transcription factors that were enriched for downstream gene targets that we identified as associated MEHP treatment using Enrichr. Across both cell types, four of the significant TFs were nuclear hormone receptors (*AR*, *ESR1*, *PPARG*, and *PPARD*) that have previously been shown to interact with phthalate metabolites. Because most of the research on the interaction between these TFs and phthalates have been performed in other cell types and tissues, it is of particular importance to note that all four of these nuclear hormone receptors have been demonstrated to be expressed in the placenta (Matsuda et al. 2013; Kim et al. 2016; Meakin et al. 2021).

While this study was the first of its kind in assessing the transcriptome of two placental cell lines in response to MEHP, there are inherent limitations in generalizing the findings to human populations. Cell line selection is an important decision when completing in vitro placental work, as each of the commercially available cell lines has strengths and weaknesses and represents a unique placental cell type and period of placental development. Several of the most common placental cell lines (BeWo, JEG-3, JAR) are derived from choriocarcinomas which may not be reflective of normal placental function and genetics (Bačenková et al. 2022). The immortalized placental cell line HTR-8/SVneo used in this study represents a first trimester extravillous trophoblast phenotype, while the primary cells represent term syncytiotrophoblast cells with known fetal sex. Given that most epidemiological studies of the placenta are performed in bulk tissue, in vitro assessments of the placenta are critical to understanding the unique roles and responses of placental trophoblast subtypes to environmental exposures. Future research should aim to expand upon the use of primary placental tissue culture, particularly in early pregnancy to understand the intricacies and interplay of cell types present during the crucial developmental window and how they may be altered in response to toxicants.

Conversely, in vitro exposure studies introduce limitations not inherent to epidemiological studies, which include the use of standalone chemicals rather than mixtures and the need to select an environmentally relevant dose while taking into consideration differing pathways of exposure and metabolism for each in vitro model. In this study, we treated cells with the monoester metabolite (MEHP) of parent phthalate compound DEHP, as phthalates are known to undergo rapid metabolism in vivo (Zhang et al. 2021), meaning that placental cells in vivo are more likely to be exposed to the metabolite than the parent compound. The two higher MEHP concentrations (90 µM and 180 µM) were selected based on previous in vitro studies of MEHP in placental cell lines (Tetz et al. 2013, 2015; Meruvu et al. 2016a, b) (Online Resource 2). These concentrations are much higher than MEHP concentrations that have been measured in human urine or cord blood (Li et al. 2013; Maekawa et al. 2017). Acute exposure studies, such as the one performed in this paper with only a 24-h exposure length, may need higher than average levels of phthalates to account for the continuous exposure seen in humans. We also selected a lower concentration (1 µM MEHP), which was in line with concentrations used in at least two previous cell studies (Wang et al. 2016; Gao et al. 2017) that also fell in the range of concentration values (1.1 × 10^–4^ µM to 2.2 µM) measured in maternal urine in the CANDLE cohort (Paquette et al. 2021). Using a lower, more biologically relevant dose is of particular importance for phthalates, as they have been shown to exhibit a non-monotonic dose response that have at times been below the published no observed adverse effect level (NOAEL), underscoring the importance of low-dose phthalate concentrations in experimental research (Hill et al. 2018). Phthalate concentrations can differ substantially between maternal urine and placental measurements. A recent analysis of a subset of CANDLE participants (*N* = 50) revealed that concentrations of MEHP quantified within the placenta were up to 800 times higher than average urinary MEHP across the second and third trimesters, and MEHP was the phthalate metabolite with the largest discrepancy between urine and placenta (Liang et al. 2022). This study highlights the importance of selecting a wide range of concentrations for in vitro analyses*.* Analysis of the placental transcriptome in response to chemical mixtures, to more accurately model human exposures, is an area of research that needs more attention in both epidemiological and in vitro studies (Lapehn and Paquette 2022).

Although similarities were identified in DEGs and KEGG pathways for the two placental cell types (HTR-8/SVneo and primary syncytiotrophoblasts), overall, there were more differences. Similarly, there were only a small number of overlapping DEGs identified between this study and the CANDLE study. These differences may be attributable to differences in trophoblast phenotype and/or trimester of origin for sampling or exposure data. The CANDLE study evaluated phthalate concentrations in maternal urine in the second and third trimesters and the associated placental gene expression changes in bulk tissue from term placentas finding more differences overall with respect to second trimester phthalate concentrations and little overlap in DEGs across each time point (Paquette et al. 2021). These findings potentially highlight the second trimester as being a more vulnerable window of time for phthalate exposure (Paquette et al. 2021). The two cell types used in this in vitro assessment of MEHP represented both different trophoblast phenotypes (extravillous trophoblast vs. syncytiotrophoblast) and different trimesters of origin (1st trimester vs 3rd trimester (term)). The primary syncytiotrophoblast cells from term placentas showed lower sensitivity to MEHP based on the total number of DEGs, similar to findings from the CANDLE study, which could suggest decreased transcriptional response at this time point. Future analyses (potentially in animal models) would benefit from evaluating the same trophoblast phenotype across all three trimesters with matched exposure data to elucidate whether measured differences in gene expression are due to vulnerable windows of exposure in specific trimesters or due to differences in trophoblast phenotype and function. This is not currently feasible through in vitro approaches or within human studies. Given the differences in phthalate-induced gene expression changes across these two cell models, we believe that single-cell RNA sequencing of the placenta should be prioritized when feasible. Under circumstances where single-cell approaches are not amenable, we recommend surrogate variable analysis (Leek 2014) or other cellular deconvolution approaches to account for cellular heterogeneity of bulk tissue samples (Campbell et al. 2021).

Overall, the results of this study highlight that phthalate metabolite MEHP causes changes to the placental transcriptome that are dependent on placental trophoblast subtype, MEHP concentration, and fetal sex. We identified three genes that were associated with MEHP exposure in both cell and human studies, including an lncRNA transcript (*NEAT1*) that is involved in transcriptional regulation through paraspeckle formation (Li et al. 2017), and has been shown to promote expression of inflammatory genes (Zhang et al. 2019). This highlights the need to better characterize the roles of these transcripts in human health. Transcription factors with known phthalate interactions (*PPARG*, *PPARD*, *AR*, *ESR1*) were reported as enriched for both cell types. Beyond these primary findings, this study has also generated an extensive list of mRNAs, lncRNAs, and pathways that are significantly affected by phthalates and should be evaluated through further candidate gene studies. These genes and pathways should be evaluated in particular for potential roles in adverse pregnancy and early life health outcomes. Linking placental gene expression to exposures and outcomes would allow for chemical monitoring and toxicological risk assessment that could eventually lead to identification of placental biomarkers of phthalate toxicity. Additionally, future work should evaluate the role of nuclear hormone receptor TFs in eliciting altered gene expression through identifying genomic binding sites following exposure to MEHP.

## Supplementary Information

Below is the link to the electronic supplementary material.

Supplementary file1 (PDF 148 KB) Online Resource 1 Representative image of primary trophoblast cells (Male) at 24 hours (a), 48 hours (b), and 72 hours (c). Syncytialization progresses spontaneously and can be noted by the fused cells in images b and c compared to the independent cells that are not aggregated in image a. Syncytialization was confirmed visually at 48 hours prior to treating with DMSO or phthalates from 48 to 72 hours

Supplementary file2 (DOCX 31 KB) Online Resource 2 Table of recent studies of in vitro phthalate exposure in placental cell lines that highlights length of exposure, phthalate metabolites, and phthalate concentration

Supplementary file3 (XLSX 860 KB) Online Resource 3 Table of differentially expressed genes (DEGs) from each cell type and MEHP treatment group

Supplementary file4 (XLSX 22 KB) Online Resource 4 Full list of significant (FDR<0.05) KEGG pathways for 90µM and 180µM MEHP in HTR-8/SVneo cells. Pathway gene number indicates the size of the KEGG pathway

Supplementary file5 (XLSX 17 KB) Online Resource 5 All significant (FDR<0.05) transcription factors from the Enrichr analysis in HTR-8/SVneo cells sorted by average FDR across doses. Total downstream genes indicate the total number of genes downstream of the transcription factor, whereas DEG number indicates the number of significant genes from a dose group found to be downstream of that TF

Supplementary file6 (XLSX 14 KB) Online Resource 6 KEGG pathways (p<0.05) for MEHP from the 2nd and 3rd trimester CANDLE study compared to significant KEGG pathways (FDR<0.05) for HTR-8/SVneo and primary syncytiotrophoblasts exposed to 90µM or 180µM MEHP

## Data Availability

RNA sequencing data is available in the NCBI Gene Expression Omnibus (GEO) database under accession number GSE217210. The code associated with this project is publicly available on GitHub (https://github.com/SLapehn/PlacentalCell_MEHP_RNAseq).
