# Supplementary material for: Mono(2-ethylhexyl) phthalate induces transcriptomic changes in placental cells based on concentration, fetal sex, and trophoblast cell type
Source: Arch Toxicol. 2023 Jan 25;97(3):831–47. doi: 10.1007/s00204-023-03444-0 (PMC9968694; doi:10.1007/s00204-023-03444-0)
Supplement: Supplementary file 1 — Supplementary file1 (PDF 148 KB) Online Resource 1 Representative image of primary trophoblast cells (Male) at 24 hours (a), 48 hours (b), and 72 hours (c). Syncytialization progresses spontaneously and can be noted by the fused cells in images b and c compared to the independent cells that are not aggregated in image a. Syncytialization was confirmed visually at 48 hours prior to treating with DMSO or phthalates from 48 to 72 hours [file 204_2023_3444_MOESM1_ESM.pdf]

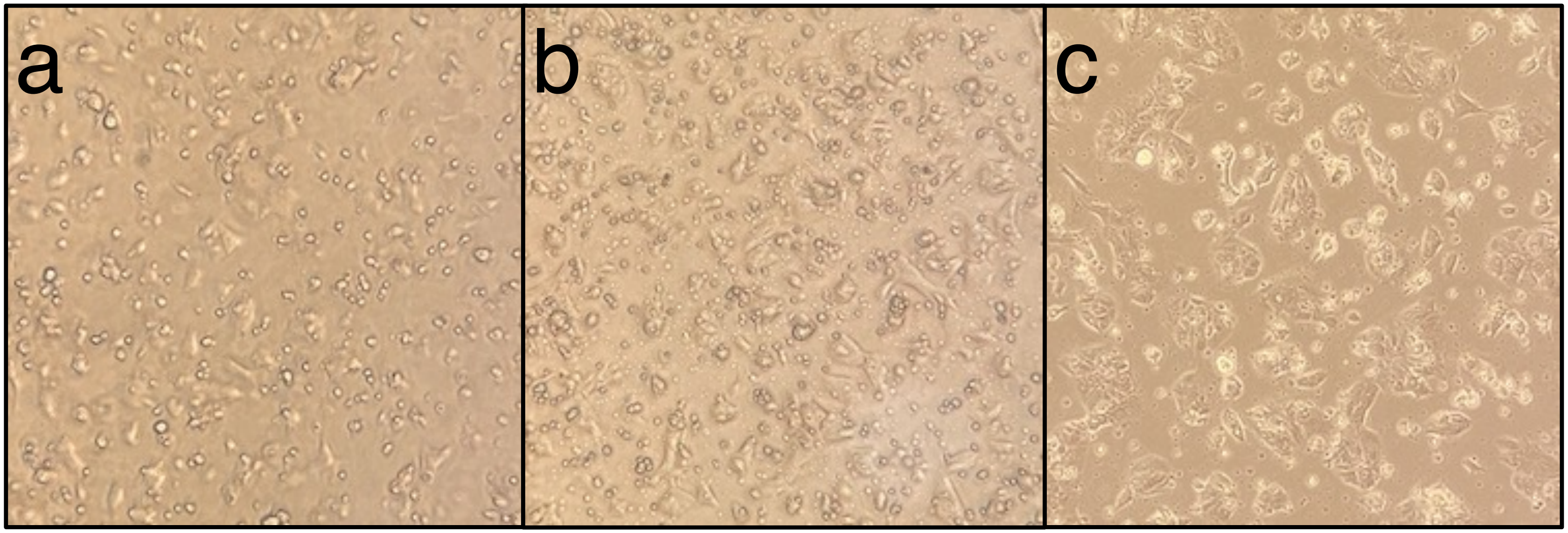

Paper Details:
Mono(2-ethylhexyl) phthalate induces transcriptomic changes in placental cells based on concentration, fetal sex, and trophoblast cell type

Samantha Lapehn, Scott Houghtaling, Kylia Ahuna, Leena Kadam, James W. MacDonald, Theo K. Bammler, Kaja Z. LeWinn, Leslie Myatt, Sheela Sathyanarayana, Alison G. Paquette

Corersponding Author Email: alison.paquette@seattlechildrens.org
Corresponding Author Affiliations: Seattle Children's Research Institute and University of Washington
