# Supplementary material for: Mono(2-ethylhexyl) phthalate induces transcriptomic changes in placental cells based on concentration, fetal sex, and trophoblast cell type
Source: Arch Toxicol. 2023 Jan 25;97(3):831–47. doi: 10.1007/s00204-023-03444-0 (PMC9968694; doi:10.1007/s00204-023-03444-0)
Supplement: Supplementary file 2 — Supplementary file2 (DOCX 31 KB) Online Resource 2 Table of recent studies of in vitro phthalate exposure in placental cell lines that highlights length of exposure, phthalate metabolites, and phthalate concentration [file 204_2023_3444_MOESM2_ESM.docx]

| **Citation** | **Cell Type** | **What Concentration?** | | | | | | | | | **Phthalate** | | | | | | | | | | | | **Length of Exposure (hrs)** | | | | | | | | |
| --- | --- | --- | --- | --- | --- | --- | --- | --- | --- | --- | --- | --- | --- | --- | --- | --- | --- | --- | --- | --- | --- | --- | --- | --- | --- | --- | --- | --- | --- | --- | --- |
|  |  | ***<10 µM*** | ***10-20 µM*** | ***20-50 µM*** | | ***50-100 µM*** | ***100-150***  ***µM*** | ***150-200 µM*** | | ***>200 µM*** | ***DEHP*** | ***MEHP*** | ***EHA*** | ***DMP*** | ***BBP*** | ***DBP*** | ***DPP*** | ***BBOP*** | ***DCHP*** | ***DHP*** | ***DNOP*** | ***DINP*** | ***1*** | ***2*** | ***4*** | ***8*** | ***12*** | ***24*** | ***48*** | ***72*** | ***7d*** |
| (Xu 2005) | HRP-1 |  |  | 25 | | 50 | 100 | 200 | |  | X | X | X |  |  |  |  |  |  |  |  |  |  | X | X | X | X | X |  |  |  |
| (Tetz et al. 2013) | HTR-8/SVneo |  | 11.25 | 22.5 | | 45, 90 |  | 180 | |  |  | X |  |  |  |  |  |  |  |  |  |  | X |  | X | X |  |  | X |  |  |
| (Meruvu et al. 2016b) | HTR-8/SVneo |  | 10 | 25 | | 50 | 100 | 180 | |  |  | X |  |  |  |  |  |  |  |  |  |  |  | X | X | X |  | X | X | X |  |
| (Xu et al. 2006) | HRP-1 |  |  |  | | 50 |  |  | |  | X | X | X |  |  |  |  |  |  |  |  |  |  |  |  |  |  | X |  |  |  |
| (Tetz et al. 2015) | THP-1 and placental macrophages |  | 10 |  | | 45, 90 |  | 180 | | 360 |  | X |  |  |  |  |  |  |  |  |  |  |  |  | X | X |  | X | X |  |  |
| (Meruvu et al. 2016a) | HTR-8/SVneo |  |  | 25 | | 50 | 100 | 180 | |  |  | X |  |  |  |  |  |  |  |  |  |  |  |  | X |  |  | X | X |  |  |
| (Wang et al. 2016) | Primary cytotrophoblasts | 1 | 10 |  | | 50 |  | 150 | | 300, 500 |  | X |  |  |  |  |  |  |  |  |  |  |  |  |  |  |  | X |  |  |  |
| (Xu et al. 2016) | JEG-3 |  |  |  | |  | 100 |  | |  |  | X |  |  |  | X | X | X | X | X | X | X |  |  |  |  | X |  |  |  |  |
| (Gao et al. 2017) | HTR-8/SVneo | 1 | 10 |  | |  | 100 |  | |  |  | X |  |  |  |  |  |  |  |  |  |  |  |  |  |  |  | X |  |  |  |
| (Pérez-Albaladejo et al. 2017) | JEG-3 | 0.01,  0.1, 1 | 10 |  |  | | 100 | |  | 1000 | X |  |  | X | X | X |  |  |  |  |  |  |  |  |  |  |  | X |  |  |  |
| (Shoaito et al. 2019) | Primary term Cytotrophoblasts | 0.1, 1 | 10 |  | | 50 | 100 |  | | 500 |  | X |  |  |  |  |  |  |  |  |  |  |  |  |  |  |  | X |  |  |  |
| (Petit et al. 2018) | JEG-3 | 1, 5 | 10 | 15, 25 | |  |  |  | |  | X | X |  |  |  |  |  |  |  |  |  |  |  |  |  |  |  |  |  | X |  |
| (Zhang et al. 2020) | JEG-3 |  | 20 |  | |  |  | 200 | | 500 | X | X |  |  |  |  |  |  |  |  |  |  |  |  |  |  |  |  |  |  | X |
| (Du et al.  2020) | HTR-8/SVneo and JEG-3 | 4 |  | 40 | |  | 100 |  | | 400 | X |  |  |  |  |  |  |  |  |  |  |  |  |  | X |  | X | X |  |  |  |

**Online Resource 2** Table of recent studies of in vitro phthalate exposure in placental cell lines that highlights length of exposure, phthalate metabolites, and phthalate concentration.

**Title:** Mono(2-ethylhexyl) phthalate induces transcriptomic changes in placental cells based on concentration, fetal sex, and trophoblast cell type

**Authors:** Samantha Lapehn, Scott Houghtaling, Kylia Ahuna, Leena Kadam, James W. MacDonald, Theo K. Bammler, Kaja Z. LeWinn, Leslie Myatt, Sheela Sathyanarayana, Alison G. Paquette

**Corresponding Author Email:** [**alison.paquette@seattlechildrens.org**](mailto:alison.paquette@seattlechildrens.org)

**Corresponding Author Affiliations:** Seattle Children’s Research Institute, University of Washington
